# The Mysterious Diver: A Case Report of a Giant Esophageal Fibrovascular Polyp With Intermittent Oral Cavity Prolapse

**DOI:** 10.7759/cureus.109600

**Published:** 2026-05-25

**Authors:** Afrah Fathima Karimbanakkal Edakkattu, Abdul Salam RT

**Affiliations:** 1 Internal Medicine, Government Medical College Manjeri, Kerala University of Health Sciences, Manjeri, IND; 2 ENT, Government Medical College Manjeri, Kerala University of Health Sciences, Manjeri, IND

**Keywords:** dysphagia, esophageal tumors, fibrovascular polyp, lateral pharyngotomy, transoral prolapse

## Abstract

Fibrovascular polyps of the esophagus are uncommon, benign intraluminal growths. Their gradual growth and vague symptoms often lead to delays in diagnosis, placing patients at risk for serious complications such as sudden airway obstruction caused by regurgitation. We present a case involving a 56-year-old woman who experienced a two-year history of a foreign body sensation in her throat and a six-month history of an intermittently protruding mass in her mouth. Despite numerous consultations and several indirect laryngoscopic examinations, no abnormalities were identified, resulting in a delayed diagnosis. Dynamic videolaryngoscopy performed during the gag reflex revealed a smooth pedunculated mass protruding into the oral cavity from the right side. Imaging studies showed dilatation of the upper and middle esophagus along with a long intraluminal soft tissue mass. Flexible upper gastrointestinal endoscopy revealed a large polypoidal lesion with a long pedicle originating from the right lateral wall just above the cricopharynx and extending into the esophagus. Surgical treatment was carried out using a combined approach involving tracheostomy, direct hypopharyngoscopy, and left lateral pharyngotomy, allowing safe identification, ligation, and complete removal of the mass. Histopathological analysis confirmed the presence of a giant fibrovascular polyp. The postoperative recovery was uneventful, and the patient remains symptom-free during follow-up. This case emphasizes the diagnostic challenges associated with giant fibrovascular polyps and highlights the importance of dynamic assessment and prompt surgical intervention to prevent life-threatening complications.

## Introduction

Benign tumors of the esophagus are uncommon, accounting for less than 1% of all esophageal neoplasms, and are broadly classified into intramural, extramucosal, and intraluminal lesions [1]. Among these, intramural tumors such as leiomyomas are most frequently encountered, whereas intraluminal lesions are distinctly infrequent [2]. Fibrovascular polyps are the most common subtype within the intraluminal category, despite their low overall incidence. These lesions arise from the submucosa and are typically covered by intact stratified squamous epithelium [3].

Fibrovascular polyps are characteristically slow-growing and pedunculated, most often originating from the cervical esophagus near the cricopharyngeal region [4]. Histologically, they consist of variable proportions of fibrous tissue, adipose tissue, and vascular structures, which has led to the use of multiple terminologies in the literature, including fibrolipoma, fibroma, and fibroepithelial polyp [5]. Because of their indolent growth pattern and intraluminal location, symptoms are often vague and nonspecific in the early stages, commonly manifesting as globus sensation or mild dysphagia [6]. As the lesion enlarges, patients may develop progressive dysphagia, regurgitation of the mass into the oral cavity, or respiratory symptoms. In advanced cases, sudden regurgitation of a large polyp has been associated with acute airway obstruction and fatal asphyxiation [7].

Diagnosis is frequently delayed, as routine or static examinations of the larynx and hypopharynx may appear normal when the lesion remains within the esophageal lumen [8]. Dynamic assessment using videolaryngoscopy, along with endoscopic and radiological evaluation, plays a crucial role in identifying these lesions and determining their extent and point of attachment [9]. Surgical excision remains the definitive treatment, with the choice of approach guided by the size of the polyp, length and vascularity of the pedicle, and anatomical accessibility [10]. The present report describes a case of a giant cervical esophageal fibrovascular polyp presenting with intermittent oral cavity prolapse, highlighting the diagnostic challenges, importance of dynamic evaluation, and rationale for definitive surgical management.

## Case presentation

A 56-year-old Indian woman of South Asian ethnicity presented to the Department of Otorhinolaryngology, Government Medical College Manjeri, Manjeri, Kerala, India, with a two-year history of persistent globus sensation in the throat. The symptom was insidious in onset, nonpainful, and initially nonprogressive. Over the preceding six months, she experienced intermittent prolapse of a mass into the oral cavity, most noticeable during gagging or retching, with spontaneous reduction thereafter. There was no associated odynophagia, weight loss, voice change, hemoptysis, choking episodes, or dyspnea at initial presentation. She had no significant past medical or surgical history, was not on long-term medications, and reported no relevant family history. Social history was noncontributory. Prior to referral, she had consulted multiple healthcare centers, where indirect laryngoscopy was performed when the mass was not actively prolapsing and was repeatedly reported as normal; therefore, no definitive diagnosis was established (Table 1). 

**Table 1:** Timeline of clinical events. ENT: ear, nose, and throat, SARS-CoV-2: severe acute respiratory syndrome coronavirus 2, GI: gastrointestinal, POD-3: postoperative day 3.

Time Point	Clinical Event
2 years prior	Onset of persistent globus sensation
6 months prior	Intermittent prolapse of mass into oral cavity
Multiple visits	Indirect laryngoscopy reported as normal
Initial ENT evaluation	Dynamic prolapse noted on tongue base palpation and videolaryngoscopy
Imaging	Dilated upper/middle esophagus with 7.4-cm intraluminal lesion
Planned intervention	Endoscopic excision planned
~1 month gap	Lost to follow-up due to SARS-CoV-2 pandemic
Re-presentation	Progressive dysphagia (solids → liquids); no oral prolapse
Upper GI endoscopy	Polypoid lesion above cricopharynx with 27-cm pedicle
Surgery	Tracheostomy + hypopharyngoscopy + left lateral pharyngotomy
Postoperative period	Uneventful recovery; decannulation on POD-3
Follow-up	Asymptomatic, no recurrence

Clinical findings

**Figure 1:**
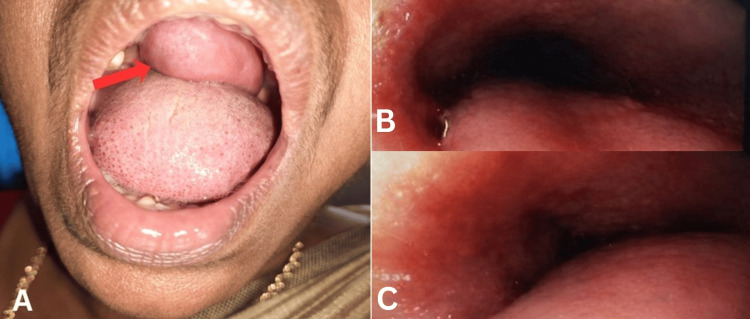
(A) Clinical photograph showing the intermittent prolapse of a smooth, pedunculated mass into the oral cavity during gagging, suggesting an intraluminal lesion. (B) Flexible upper gastrointestinal endoscopy showing a large polypoidal lesion arising from the right lateral wall just above the cricopharynx. (C) Flexible upper gastrointestinal endoscopy demonstrating the long, flat pedicle of the fibrovascular polyp extending distally into the esophagus.

On general and systemic examination, the patient was stable. Examination of the oral cavity and oropharynx was unremarkable, and neck examination revealed no palpable masses or lymphadenopathy. Indirect laryngoscopy demonstrated normal supraglottic, glottic, and hypopharyngeal structures. However, digital palpation of the base of the tongue elicited prolapse of a smooth, firm mass into the oral cavity, raising suspicion of a dynamic intraluminal lesion (Figure 1). 

**Figure 2:**
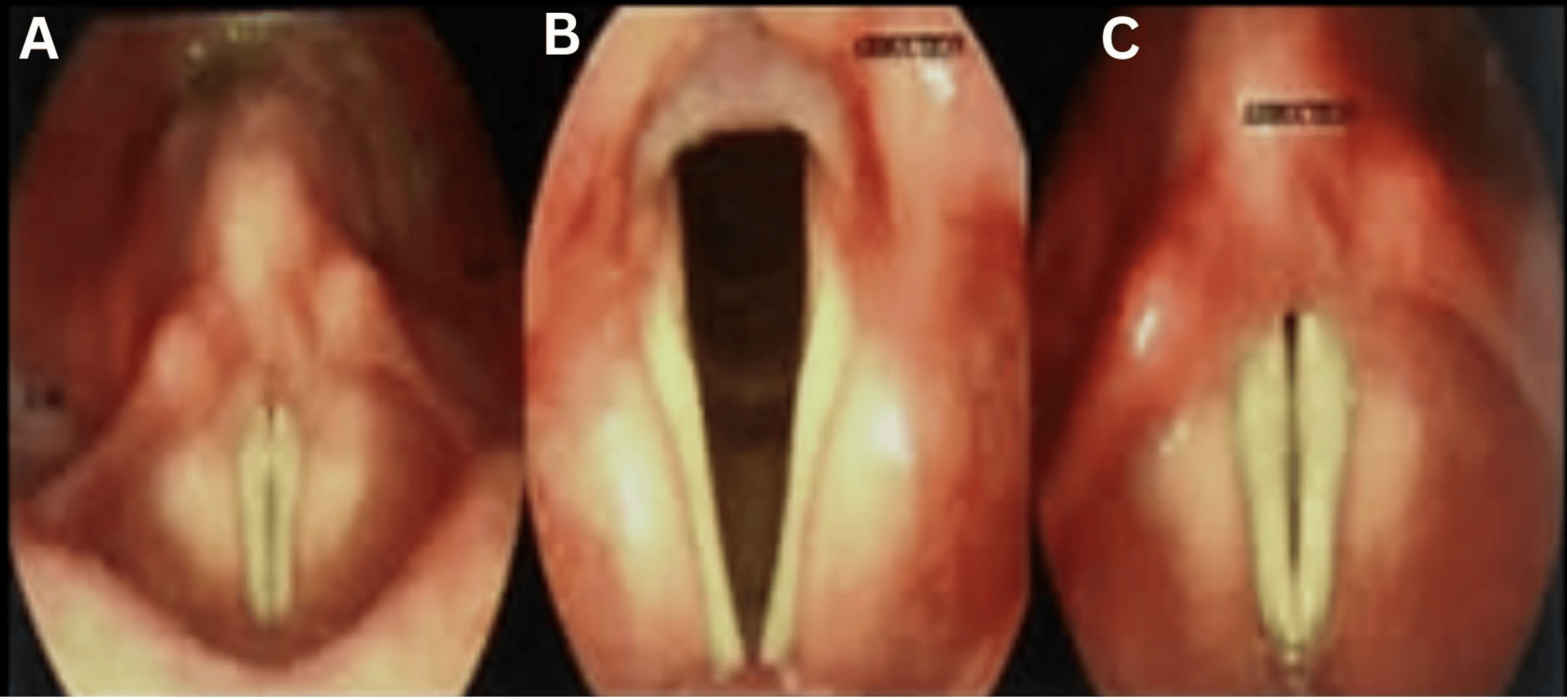
Videolaryngoscopic images. (A) Normal laryngeal and hypopharyngeal anatomy at rest. (B, C) On eliciting the gag reflex, a smooth, pedunculated mass is seen prolapsing into the oral cavity from the right side; the site of attachment is not visualized.

Flexible videolaryngoscopy performed at rest showed normal laryngeal and hypopharyngeal anatomy. When the gag reflex was elicited, a smooth, pedunculated mass was observed prolapsing into the oral cavity from the right side, although the site of attachment could not be visualized (Figure 2).

Diagnostic assessment

**Figure 3:**
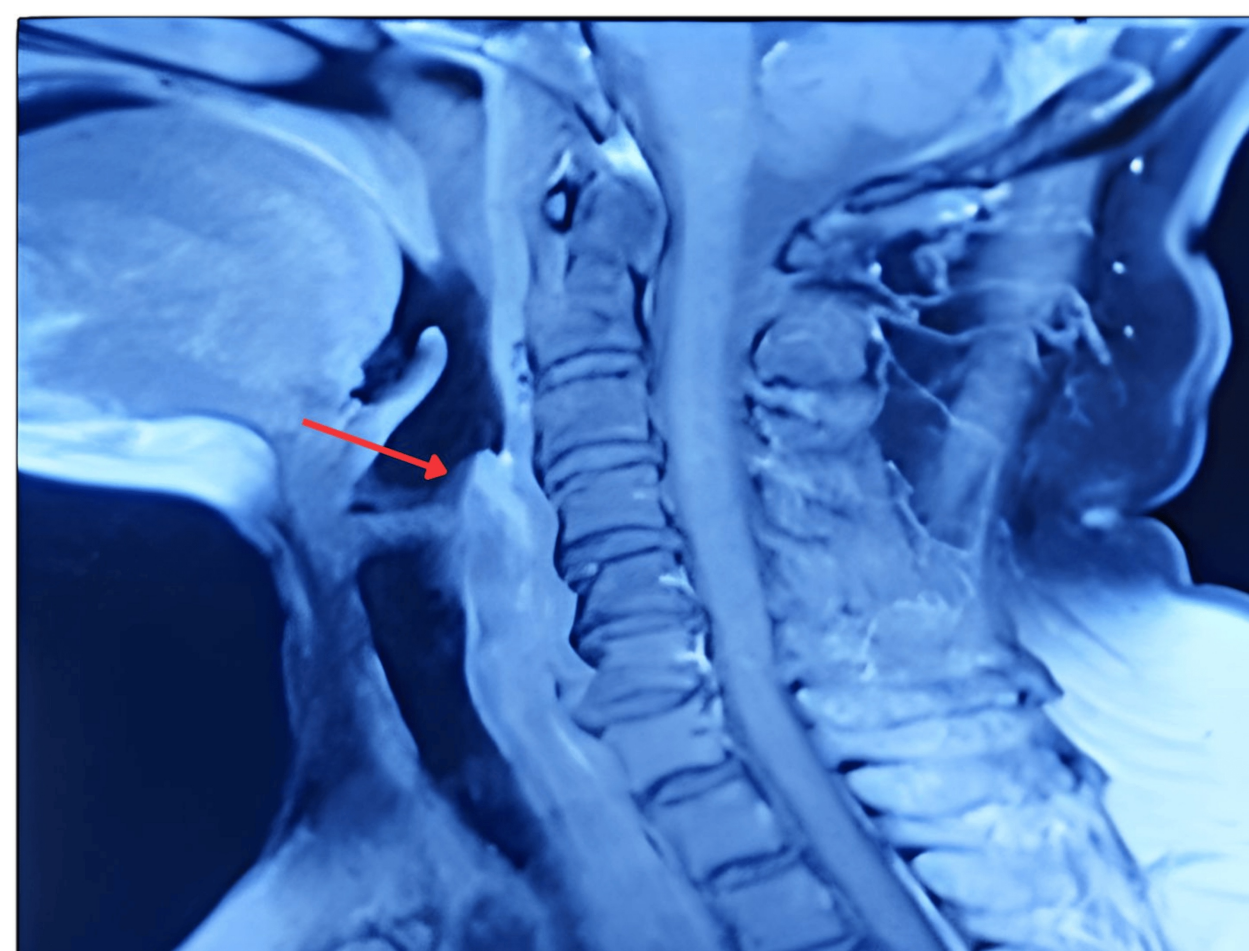
Radiological imaging of the esophageal polyp. Radiological image showing upper and middle esophageal dilatation with a long intraluminal soft-tissue lesion extending inferiorly to the level of the tracheal bifurcation. The arrow indicates the intraluminal polyp.

Radiological evaluation demonstrated dilatation of the upper and middle esophagus with a 7.4-cm intraluminal soft-tissue lesion extending inferiorly to the level of the tracheal bifurcation. Based on these findings, endoscopic evaluation and excision were planned. The patient was subsequently lost to follow-up due to the SARS-CoV-2 pandemic. She re-presented approximately one month later with worsening dysphagia, initially for solids and later for liquids. At this stage, the previously observed oral cavity prolapse was no longer demonstrable, even with gagging. Flexible upper gastrointestinal endoscopy revealed a large polypoidal lesion located just above the cricopharynx on the right lateral wall, with a long, flat pedicle measuring approximately 27 cm, extending distally into the esophagus (Figure 3).

Differential diagnoses 

Fibrovascular polyp, esophageal lipoma, pedunculated leiomyoma, and other benign intraluminal esophageal tumors. The presence of a long pedicle, intraluminal growth pattern, cervical esophageal origin, and imaging characteristics favored a fibrovascular polyp over other entities. Pre-operative biopsy was not attempted due to the risk of hemorrhage and airway compromise.

Therapeutic intervention

**Figure 4:**
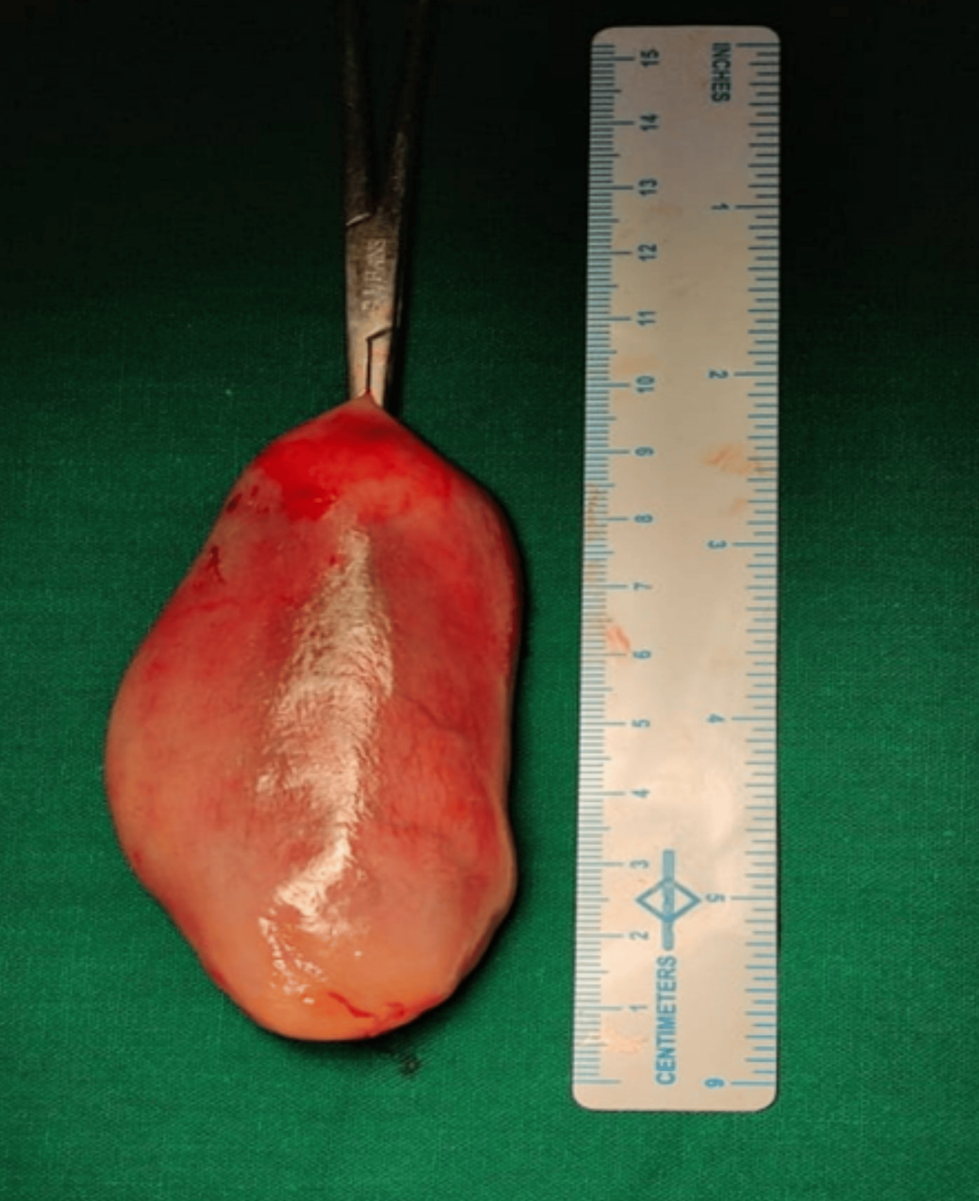
Gross specimen of the excised fibrovascular polyp measuring 12 × 5 cm, showing a smooth, elongated, and firm pedunculated mass.

Definitive surgical management was undertaken under general anesthesia. A preliminary tracheostomy was performed to secure the airway, followed by direct hypopharyngoscopy. Due to the size and length of the lesion, endoscopic retrieval was unsuccessful. A left lateral pharyngotomy was therefore performed. The mass was identified within the cervical esophagus. The pedicle and feeding vessels were visualized at the right lateral wall of the cricopharynx, ligated, and the lesion was excised in toto. The excised specimen measured 12 × 5 cm, appeared smooth and firm, and was sent for histopathological examination (Figure 4).

Follow-up and outcomes

**Figure 5:**
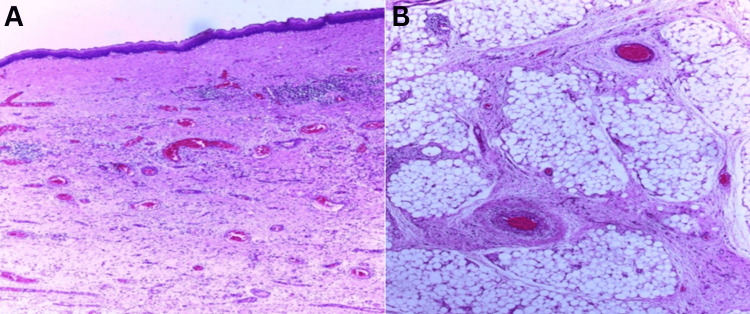
Histopathological examination. (A) Low-power view showing a polyp lined by stratified squamous epithelium with underlying fibrovascular stroma (H&E stain). (B) High-power view demonstrating congested blood vessels and lobules of adipose tissue separated by fibrovascular septae, consistent with a fibrovascular polyp (H&E stain).

Postoperatively, nasogastric feeding was initiated on the first postoperative day. The tracheostomy tube was decannulated on postoperative day three, and enteral feeding was continued for one week before resumption of oral intake. The postoperative course was uneventful. Histopathological examination demonstrated a polyp lined by stratified squamous epithelium, with underlying fibrovascular stroma containing congested blood vessels and lobules of adipose tissue separated by fibrovascular septae, confirming the diagnosis of a giant fibrovascular polyp (Figure 5).

## Discussion

This report describes a case of a giant cervical esophageal fibrovascular polyp presenting with prolonged globus sensation and intermittent oral cavity prolapse, ultimately progressing to dysphagia. The case highlights the diagnostic challenges posed by intraluminal esophageal lesions with dynamic behavior and underscores the importance of appropriate diagnostic strategies and definitive surgical management. Fibrovascular polyps constitute approximately 0.5%-1% of benign esophageal tumors and are the most common intraluminal benign lesions of the esophagus despite their overall infrequency [1]. They typically arise from the cervical esophagus near the cricopharyngeal region, where repetitive swallowing-related traction on redundant submucosal tissue contributes to progressive elongation and intraluminal growth [2]. The characteristic pedunculated morphology allows these lesions to attain considerable length while remaining asymptomatic for extended periods.

Clinical presentation is often delayed because of nonspecific early symptoms. Dysphagia is the most common complaint, followed by regurgitation and respiratory symptoms [3]. Intermittent prolapse of the lesion into the oral cavity, as observed in this case, is a recognized feature, although inconsistently reported. Schuhmacher et al. noted that oral regurgitation may diminish as the lesion enlarges and migrates distally within the esophagus, which may explain the disappearance of this sign during later stages in some patients [4]. This dynamic behavior contributes to diagnostic difficulty and may result in repeated normal findings on routine examinations. Routine indirect laryngoscopy frequently fails to identify fibrovascular polyps when the lesion remains intraluminal, leading to underdiagnosis or misattribution of symptoms [5]. In this case, repeated normal indirect laryngoscopic examinations delayed diagnosis. Dynamic assessment using videolaryngoscopy during the gag reflex proved critical in raising suspicion, supporting prior observations that static examinations alone may be insufficient [2]. Radiological investigations such as barium swallow, computed tomography, and magnetic resonance imaging assist in delineating the lesion’s extent and composition, whereas endoscopy is essential for identifying the pedicle and planning the intervention [2,6].

Surgical excision remains the definitive treatment. Endoscopic resection may be appropriate for small lesions with thin pedicles, typically less than 2 cm in diameter. However, giant fibrovascular polyps often require open or combined surgical approaches because of their size, vascularity, and the risk of incomplete excision or hemorrhage [1]. In this case, endoscopic retrieval was unsuccessful because of the lesion size and pedicle length, necessitating a lateral pharyngotomy. Open transcervical approaches provide superior exposure and allow safe identification and ligation of the vascular pedicle, minimizing intraoperative complications [6,7]. Complete excision is essential to prevent recurrence, which, although uncommon, has been reported following incomplete removal [3]. Of particular clinical importance is the potential for sudden regurgitation of large fibrovascular polyps, which can lead to acute airway obstruction and fatal asphyxiation, underscoring the need for timely diagnosis and definitive management [5].

The principal strength of this case lies in its clear demonstration of diagnostic delay due to intermittent symptoms and normal routine examinations, highlighting the value of dynamic assessment. Limitations include the single-case nature and relatively short follow-up period. Nevertheless, this case reinforces important diagnostic and therapeutic principles relevant to clinical practice.

## Conclusions

This case illustrates that a giant esophageal fibrovascular polyp may present with prolonged globus sensation, intermittent oral cavity prolapse, and later progressive dysphagia, whereas routine laryngeal examination at rest may remain normal. In patients with a history of intermittent oral mass prolapse, dynamic assessment during gagging or retching can provide an important diagnostic clue.

Accurate localization of the lesion, pedicle, and site of attachment is important for treatment planning. Because large mobile polyps may pose a risk of airway compromise, timely definitive excision should be considered once the diagnosis is established. In the present case, a combined surgical approach allowed safe pedicle control and complete removal, with an uneventful postoperative recovery.
